# HIV/Aids and COVID-19 in Brazil: in four decades, two antithetical approaches to face serious pandemics

**DOI:** 10.1590/0074-02760210071

**Published:** 2021-06-21

**Authors:** Bernardo Galvão-Castro, Maria Fernanda Rios Grassi, Euclides Ayres de Castilho, Dirceu Bartolomeu Greco

**Affiliations:** 1 Escola Bahiana de Medicina e Saúde Pública, Curso de Medicina, Salvador, BA, Brasil; 2 Fundação Oswaldo Cruz-Fiocruz, Instituto Gonçalo Moniz, Laboratório Avançado de Saúde Pública, Salvador, BA, Brasil; 3 Universidade de São Paulo, Faculdade de Medicina, Departamento de Medicina Preventiva, São Paulo, SP, Brasil; 4 Universidade Federal de Minas Gerais, Faculdade de Medicina, Departamento de Clínica Médica, Belo Horizonte, MG, Brasil

**Keywords:** HIV/AIDS, Covid-19, pandemic, Brazil

## Abstract

In the space of four decades, Brazil has faced two serious pandemics: human immunodeficiency virus/acquired immunodeficiency syndrome (HIV/AIDS) and Coronavirus disease 2019 (COVID-19). The country’s response to HIV/AIDS was coordinated by several stakeholders and recognised the importance of scientific evidence in guiding decision-making, and a network offering monitoring and antiretroviral treatment was provided through coordinated efforts by the country’s universal health system. Conversely, the lack of a centrally coordinated strategy and misalignment between government ministries regarding the COVID-19 pandemic response, together with the denial of scientific evidence, promotion of ineffective treatments and insufficient vaccination efforts, have all led to the uncontrolled spread of infection, the near-total collapse of the health system and excess deaths.

In 1982, Brazil reported its first cases of acquired immunodeficiency syndrome (AIDS) as the country was emerging from a military dictatorship (1964-1985). In 1986, the Ministry of Health (MoH) established the National STD/AIDS Program, which involved extensive stakeholder participation.^1^ These stakeholders included the Oswaldo Cruz Foundation (Fiocruz), one of the most important scientific and technological institutions in Latin America, non-governmental organisations (NGOs), public universities, and state/municipal health secretariats. The program’s initiatives, based on scientific knowledge, respect for human rights and the opposition of discrimination against people living with human immunodeficiency virus (HIV)/AIDS, led to the recognition of Brazil as a model among low- and middle-income countries. These included, but were not limited to, mandatory screening for bloodborne infectious diseases and the prohibition of paid blood donations in 1993,^2^ the establishment of nationwide specialised clinical services and the implementation of harm reduction program.^3^ In addition, national networks were implemented to monitor HIV genetic diversity,^4^ perform CD4+ T-cell counts, quantify viral load and monitor HIV drug resistance. In 1990, the Brazilian Universal Health System (SUS) was created, and in 1996 a federal law provided free access to antiretrovirals through SUS.^5^ Morbidity and mortality decreased, and while infection rates fell to 0.5% in the general population (2019), high prevalence continued among key populations.^6^ The comprehensive response implemented to combat the HIV/AIDS epidemic was made possible through funding provided by the Brazilian government, as well as financial support from the WHO. In addition, loans provided by the World Bank were important to organising the national response against HIV/AIDS.

HIV/AIDS and Coronavirus disease 2019 (COVID-19) are diseases with distinct epidemiological, biological and clinical profiles. However, when faced with both infections, the scientific community responded promptly by identifying and sequencing causal agents, developing diagnostic tests, characterising each disease, setting up clinical trials to evaluate potential treatments and providing clinical facilities to care for infected individuals. Concerning COVID-19, effective vaccines were quickly developed using innovative technologies. Considering its experience acquired during the AIDS pandemic, the extensive reach of SUS and the presence of a robust national immunisation program (PNI) capable of vaccinating millions per day, Brazil had all the necessary tools to effectively respond to the COVID-19 pandemic. Unfortunately, despite activating the national Emergency Health Operations Centre in January 2020, prior to diagnosing the first domestic case,^7^ the hoped-for outcomes did not materialise. Brazil’s president denied the severity of COVID-19^8^ and, lacking any scientific evidence, pressed for early treatment with chloroquine. Following the ousting of the MoH in April, his replacement agreed to push chloroquine. Government authorities have downplayed the use of masks/social distancing, and publicly denied the efficacy of developed vaccines. In addition, a 2016 law (EC95), which capped public spending for 20 years, contributed to the collapse of health systems across many cities as well as shortages in medical supplies, such as oxygen in the Amazon region.

Many factors can help explain Brazil’s antithetic response to COVID-19 in comparison to HIV/AIDS. Disparities among Brazilians throughout the continental-sised country demanded coordinated efforts in both cases. For example, the alignment between the MoH and the Ministry of Foreign Affairs enabled Brazil to have a prominent position in international forums and to express leadership in negotiations to expand universal access to antiretrovirals. In contrast, since the beginning of the COVID-19 pandemic, there has been disarticulation regarding the acquisition of inputs, such as diagnostic kits and masks, between these same ministries. The lack of appropriate strategies has also hampered the acquisition of raw materials essential to vaccine production, as well as to vaccines themselves. In addition, misguided and often negligent decisions were also taken due to deficient coordination between the MoH and the Ministry of Economy, Finance and Planning, resulting in inadequate financial support to facilitate adherence to social distancing measures.

It is important to note the autonomy of the MoH and the National AIDS Program with regard to ideological preferences or outside influence from private groups, which permitted the establishment of bold price negotiations in the purchasing of antiretrovirals, as well as the establishment of progressive health campaigns aimed at AIDS prevention. On the contrary, the government’s response to the current COVID-19 epidemic has been characterised by an absence of autonomy, as technical and expert committees have been marginalised at the expense of feeble solutions and ineffective treatments.^9^

Brazil also played an essential role to the approval to the use of compulsory licensing of drugs during pandemics, such as HIV/AIDS, as a basic human right.^10^ In contrast, the proposal for immediate suspension of issuing of patents to COVID-19 vaccines and other new technologies led for South Africa and India to the World Trade Organisation was not supported by the Brazilian government. More than 100 countries were favorable to that proposal.^11^

Denying the severity of COVID-19 and issuing recommendations for ineffective treatments has facilitated non-adherence to preventive measures.^12^ Conversely, during the AIDS pandemic, as early as 1996, the National AIDS program brought together researchers, physicians, civil society to jointly construct clinical protocols based exclusively on solid scientific evidence.^13^ The Brazilian experience with HIV/AIDS demonstrated the capability of low- and middle-income countries to treat people with equity, independently of race, gender or economic power and that this equality “seed” could be spread to other countries.

The restrictions imposed by EC95 weakened SUS and evaporated financial support necessary to spur scientific and technological development. All this is also in contrast to the AIDS epidemic measures.

All of these aspects may have potentially increased morbidity and mortality, especially among the most vulnerable populations^14^ ^,^ ^15^ ^,^ ^16^ and have hindered the government’s ability to mount an effective response against COVID-19, which could have potentially prevented hundreds of thousands of deaths. As of early April 2021, less than 5% of Brazilians had been fully vaccinated, yet the country ranks second in COVID-19 mortality with 377,000 deaths. Of note, this figure corresponds to 12% of the global mortality caused by COVID-19, despite the fact that Brazil represents just 3% of the world’s population.

The HIV/AIDS epidemic in Brazil prompted clear political decision-making and the establishment of strong partnerships among several stakeholders, including government leaders, civil society, universities, researchers and health professionals. Collaboration was also very clear and effective between ministries. As already mentioned, a good example was the alignment of the MoH and Foreign Affairs which facilitated the prominent position in various international fora, especially World Health Oraganization/Joint United Nations Programme on HIV/AIDS (WHO/UNAIDS), in defense of human rights, against discrimination and for a worldwide expansion of access to antiretrovirals. To effectively combat COVID-19, the lessons previously learned from the Brazilian experience with HIV/AIDS must be applied to counteract isolationism, boost international solidarity and cooperation, adequately finance science and quality public health services, confront anti-science rhetoric and anti-vaccine movements and prioritise egalitarian access to technological progress.^17^ ^,^ ^18^ Only through adept coordination will it be possible to address the social determinants of health that have facilitated the establishment and spread of this syndemic.^19^ Although Brazil managed to adequately confront the HIV/AIDS epidemic, which served as an example for many other countries, the lessons learned from HIV/AIDS unfortunately have not been applied in facing the COVID-19 pandemic.

## References

[1] Greco DB, Simão M (2007). Brazilian policy of universal access to AIDS treatment: sustainability challenges and perspectives. AIDS.

[2] MS (1993). Portaria 1376 de 19 de novembro de 1993. Ministério da Saúde.

[3] Mesquita F, Doneda D, Gandolfi D, Nemes MI, Andrade T, Bueno R (2003). Brazilian response to the human immunodeficiency virus/acquired immunodeficiency syndrome epidemic among injection drug users. Clin Infect Dis.

[4] Galvão-Castro B, Couto-Fernandez JC, Mello MA, Linhares-de-Carvalho MI, Castello-Branco LR, Bongertz V (1996). A nationwide effort to sistematically monitor HIV-1 diversity in Brazil preliminary results. Mem Inst Oswaldo Cruz.

[5] Victora CG, Barreto ML, Leal MC, Monteiro CA, Schmidt MI, Paim J (2011). Health conditions and health-policy innovations in Brazil the way forward. Lancet.

[6] MS (2019). Boletim epidemiológico de HIV/Aids 2019. http://www.aids.gov.br/pt-br/pub/2019/boletim-epidemiologico-de-hivaids-2019.

[7] Croda J, Oliveira WK, Frutuoso RL, Mandetta LH, Baia-da-Silva DC, Brito-Sousa JD (2020). COVID-19 in Brazil advantages of a socialized unified health system and preparation to contain cases. Rev Soc Bras Med Trop.

[8] The Lancet (2020). COVID-19 in. Brazil: "So what?". Lancet.

[9] Lasco G (2020). Medical populism and the COVID-19 pandemic. Glob Public Health.

[10] UNHCHR (2001). Access to medication in the context of pandemics such as HIV/AIDS. Commission on Human Rights Resolution.

[11] Usher AD (2020). South Africa and India push for COVID-19 patents ban. Lancet.

[12] Hallal PC (2021). SOS Brazil science under attack. Lancet.

[13] Galvão J (2002). Access to antiretroviral drugs in Brazil. Lancet.

[14] Correa-Agudelo E, Mersha T, Hernández A, Branscum AJ, MacKinnon NJ, Cuadros DF (2020). Identification of vulnerable populations and areas at higher risk of COVID-19 related mortality in the US. medRxiv.

[15] Figueiredo AM, Figueiredo DCMM, Gomes LB, Massuda A, Gil-García E, Vianna RPT (2020). Social determinants of health and COVID-19 infection in Brazil: an analysis of the pandemic. Rev Bras Enferm.

[16] Williamson EJ, Walker AJ, Bhaskaran K, Bacon S, Bates C, Morton CE (2020). Factors associated with COVID-19-related death using OpenSAFELY. Nature.

[17] Hargreaves J, Davey C, Group for lessons from pandemic HIV prevention for the COVID-19 response (2020). Three lessons for the COVID-19 response from pandemic HIV. Lancet HIV.

[18] The Lancet HIV (2021). Do not repeat mistakes from HIV in COVID-19 response. Lancet HIV.

[19] Horton R (2020). Offline COVID-19 is not a pandemic. Lancet.

